# Unraveling the forage productivity puzzle: Comparing fast and slow-growing grasses

**DOI:** 10.1371/journal.pone.0306692

**Published:** 2024-07-30

**Authors:** M. Gabriela Pittaro, Paulo G. Duchini, Gabriela C. Guzatti, André F. Sbrissia

**Affiliations:** 1 Instituto de Fisiología y Recursos Genéticos Vegetales (IFRGV), Centro de Investigaciones Agropecuarias (CIAP), Instituto Nacional de Tecnología Agropecuaria (INTA)—Unidad de Estudios Agropecuarios (UDEA), Consejo Nacional de Investigaciones Científicas y Técnicas (CONICET). Córdoba, Argentina; 2 Centro de Ciências Agroveterinárias (CAV), Universidade do Estado de Santa Catarina (UDESC), Avenida Luiz de Camões, Lages, Santa Catarina, Brazil; 3 Instituto Federal de Santa Catarina (IFSC), Campus de São Miguel, São Luiz, São Miguel do Oeste, Santa Catarina, Brazil; University of Udine: Universita degli Studi di Udine, ITALY

## Abstract

Functional traits are powerful tools for distinguishing between plants with different resource acquisition strategies. Fast-growing plants normally dominate resource-rich habitats and present trait values associated with high productivity, such as high specific leaf area (SLA), short leaf lifespan, and rapid leaf elongation rate (LER). In contrast, slow-growing species have a higher leaf weight ratio (LWR), leaf lifespan (LLS), and phyllochron, which are useful traits for survival in stressful and unfertile environments, but are normally thought to be incompatible with high productivity, even under fertile conditions. We tested the hypothesis that slow-growing forage grasses have demographic parameters (tiller population density and canopy density) that offset their slow individual traits, making them as productive as fast-growing species when grown in fertile soil. Species with contrasting growth strategies (*Arrhenatherum elatius* L. and *Festuca arundinacea Schreb* cv. Quantum II, fast and slow-growing species, respectively) were cultivated in 45 m^2^ field plots and subjected to the same cutting regime and nitrogen supply level. Functional traits and canopy attributes were continuously measured during 8 growing cycles after the establishment of the swards. *A*. *elatius* had higher SLA, LER, leaf senescence, and leaf appearance rates, whereas *F*. *arundinacea* had higher LLS and LWR values. Conversely, there were no differences in relative growth rate or forage accumulation. *F*. *arundinacea* was able to offset their plant functional traits, typically associated with slow-growing grasses, with some demographic parameter like higher tiller population density, allowing it to be as productive as the fast-growing *A*. *elatius* when both were grown in fertile soil. Therefore, we suggest cautionary use of traditional plant functional traits to explain and predict the annual productivity of slow-growing grasses.

## Introduction

Forage grasses represent a highly diverse group of plants with a global presence, thriving in both natural, undisturbed environments and highly intensified pasture-based animal production systems. These plants have undergone sophisticated evolutionary processes to adapt and persist in a multitude of contexts. It is relatively well accepted that plant traits, including functional traits, are the cornerstone of understanding (and predicting) how plants behave, grow, and interact with their surrounding environments [1].

Plant ecological schemes [2, 3] classify plants according to certain axes of specialization, each of which represents a trade-off that limits possible investments of resources to different plant organs [4]. In this context, the *Plant Economic Spectrum* (PES) concept [1, 5] provides the theoretical framework to classify plant species ranging from the ‘fast’—resource-acquisitive—trade‐off axis to species with ‘slow’- conservative life traits [6]. According to this theoretical resource acquisition scheme, plants with fast-growing traits e.g., high specific leaf area (SLA), rapid leaf extension rate (LER), high leaf nitrogen content (N), and low leaf lifespan dominate resource-rich habitats [5, 7, 8], and conservative species possess traits e.g. as high leaf dry matter content (LDMC), low tissue nutrient concentrations, and high leaf lifespan, that enhance nutrient conservation [9, 10] and increase the likelihood of plant survival under poor or infertile conditions [11–13].

Specific leaf area and LDMC are widely recognized as the most relevant traits for capturing PES [14, 15]. SLA, defined as the leaf area per unit of dry leaf biomass [16], plays a pivotal role in various leaf and plant functions such as gas exchange, Relative Growth Rate (RGR), and plant survival. It is a crucial trait for evaluating the ecological performance and productivity of plants [17] and is closely associated with canopy photosynthetic capacity [18] and leaf N content [16]. Furthermore, SLA exhibits a positive correlation with Leaf Elongation Rate (LER), a plant functional trait heavily influenced by the leaf growth zone. LER can serve as an indicator to classify grasses along a fertility gradient [19] and is normally considered a proxy for grassland productivity [20].

According to Violle et al. [18], a functional trait is defined as “any morphological, physiological, or phenological feature measurable at the individual level.” At the population level, the same authors proposed to stick the terminology used by Caswell [21] and use the "demographic parameter" instead of, for example, "population traits". Functional traits can significantly influence canopy structure (or demographic parameters), which is defined here as the distribution and arrangement of aboveground plant parts within a community [22]. For example, species with longer leaf lifespans and phyllochrons (typical slow-growing grasses) tend to have greater foliage mass per unit of ground and a higher proportion of leaves in the herbage mass [23]. A longer leaf lifespan also seems to offset the lower potential production of leaf N per unit time. The general relationship between this variable and other plant growth traits across various plant communities suggests that it is relatively universal and can serve as a common metric for ecological comparisons among diverse systems [24].

In perennial grasses, tiller population density is an important component of canopy structure [25], which is essential for the establishment and regeneration of swards [26]. Moreover, the persistence pathway of grasses with different growth strategies is linked to tiller turnover and survival throughout the year, regardless of whether they are cultivated in monocultures or mixtures [27]. Species with higher tiller survival rates (TSR) can sustain more tillers per unit area and are able to maintain greater biomass [28]. Fast-growing species possess traits that increase their capacity to capture resources (acquire nutrients), which could be a consequence, rather than the cause, of their higher relative growth rate and lower leaf lifespan (LLS) [5, 9, 29–32]. Conversely, they also present higher leaf senescence rates (LSR), which represent an important physiological process in grass growth [33]. Therefore, a higher leaf weight ratio (LWR), which is often observed in slow-growing species [34], could be advantageous for crop growth at sward levels and has been reported to be a useful indicator of potential productivity [35].

Based on the research outlined above, we investigated whether plant functional traits used to determine plant growth strategies could be reliable indicators of the potential of forage productivity and how species with contrasting growth strategies maximize their forage production in a fertile environment. We hypothesized that slow-growing grasses counteract the rapid resource acquisition traits of fast-growing grasses with some demographic parameters and that both can accumulate similar forage biomass when cultivated in nutrient-rich soil.

## Materials and methods

### Experimental area, treatments and management

This study was conducted from June 2013 to July 2015 at the Center of Agriculture and Veterinary Sciences of Santa Catarina State University, Lages, Santa Catarina, Brazil (27° 47´ S, 50° 18´ W; 960 masl). According to the Köppen classification, the region has a humid subtropical climate under oceanic influence, with cold winters, mild summers, and well-distributed rainfall throughout the year [36]. The average annual rainfall is 1,543 mm and the average temperature and radiation vary between 11°C and 857.67 Kj m^-2^ July; and 20.4˚C and 1637.46 Kj m^-2^ January. During the experimental period, no water deficit was observed throughout the year, and the average monthly temperatures were June 11.98°C, July 12.26°C, August 12.61°C, September 15.06°C, October 17.30°C, November 18.07°C, December 19.53°C (2014) and January 20.83°C, February 20.11°C, March 19.14°C, April 16.36°C, May 13.85°C, June 11.38°C (2015) (S1 Table).

To test our hypothesis, we established two potentially dominant cool-season perennial forage grasses with contrasting growth characteristics. The species were a fast-growing grass *Arrhenantherum elatius* L. cv. SCS314 Santa Vitória, and a slow-growing grass *Festuca arundinacea* Schreb. cv. Quantum II. They were seeded in June 2013 at a rate of 18 kg ha^–1^ of pure viable seeds following a completely randomized design with three replicates (45 m^2^ each) per experimental unit. The plots were seeded in June 2013 and maintained under free-growth conditions to full establishment until February 2014. The first nitrogen (N) fertilization was performed on October 27, 2013, with 70 kg N ha^-1^. In March 2014, the canopies were cut to a height of 7 cm above the soil surface, and a second N fertilization was performed at 50 kg N ha^-1^. Each time the pastures reached a height of 20 cm, they were cut 10 cm above the soil surface. The defoliation criteria were based on the sward height. A pre-cutting height of 20 cm was used because previous evaluations identified that, at this height, pastures were already intercepting 95% of the incident light for both species. This management strategy was implemented to minimize light competition. The post-cutting height was set at 10 cm above the ground level and was chosen to impose a relatively low disturbance level (defoliation of no more than 50% of the pre-cutting height). These targets were chosen to minimize the disturbance caused by defoliation and stress owing to light competition [37, 38]. The data were collected between June 2014 and April 2015. Over this period, 10 harvests were performed; we labelled cycles 1–10. Cycles 2 and 8 were discarded because of insect attacks [39] and operational issues (S2 Table).

The soil at the experimental site was a typical Inceptisol [40]. The average soil chemical characteristics (0–20 cm depth) before the experimental establishment was: pH = 4.3; organic matter = 2.1%, K = 48 mg dm^-3^, P = 3.6 mg dm^-3^, Ca = 1.16 cmol_c_ dm^–3^, Mg = 0.82 cmol_c_ dm^–3^, Al+H = 30.7 cmol_c_ dm^-3^, cation exchange capacity (CEC) at pH 7.0 = 6.27 cmol_c_ dm^-3^, base saturation = 6.4%, and clay = 52%. Based on the analysis, soil was limed to increase the pH to 6.5, and phosphorus and potassium to high to very high levels, according to the Fertilization and Liming Manual for the States of Rio Grande do Sul and Santa Catarina, Brazil (CQFS-RS/SC, 2004). Nitrogen fertilization events occurred every 40 days at 30 kg N ha^-1^ (total amount of 270 kg N ha^-1^ for the year). Nitrogen nutrition indices (NNI) were above 0.8 [33], which can be considered a threshold for good nitrogen nutrition, according to [41].

### Functional growth traits and forage productivity

Within each experimental unit (45 m^2^), 20 tillers were chosen and labelled with plastic wire and a numbered tag to evaluate individual tillers using the tissue flow technique [42]. After each harvest, 20 new tillers were chosen and labelled for subsequent measurements. All leaves in the selected tillers were measured using a ruler from the surrounding sheaths, and it was made at regular intervals throughout the cycle (every 3 to 7 days).

The leaf elongation rate (LER; cm dd^-1^) was calculated by dividing the length increment of a leaf at a specific time by the thermal time elapsed between measurements (expressed in degree-days; dd). This calculation represents the slope of the leaf elongation growth. Similarly, the leaf senescence rate (LSR; cm dd^-1^) was measured by dividing the length of senescing leaves during a specific thermal period between measurements. Patterns of LER and LSR per tiller were obtained by summing the slope elongation and senescence of the leaves within the tiller. The balance of leaf growth (BLG; cm dd^-1^) was calculated by subtracting the LER value from the LSR value for each tiller. A basal temperature of 6°C was assumed for both the grass types.

The thermal time per day was calculated as dd = [(T_MAX_ + T_MIN_)/2]-T_BASE._ where T_MAX_ is the maximum temperature per day, T_MIN_ is the minimum temperature per day, and T_BASE_ is the temperature base of each specie [43]. If the daily mean temperature (T_MAX_ + T_MIN_)/2) was less than the temperature base (T_BASE_), it was set to 0 dd, indicating no dd accumulated on that day. We summed the thermal time per day for a specific time.

The tiller extended height (cm) and length of each live leaf until the first ligule were measured. The patterns of tiller elongation rate (TER; cm dd^-1^) were recorded by dividing the length increment of the tiller during a specific time by the thermal time elapsed between measurements. For each leaf, the time of appearance (expressed in degree-days; dd) was estimated using the slope of leaf elongation and the specific thermal time elapsed. The phyllochron (Ph; dd^-1^) of each leaf was estimated by considering the thermal time between the appearance of two sequential leaves.

The number of leaves per tiller (Ln) was measured using an adapted [44] system for wheat (*Triticum aestivum* L. em. Thell.). Briefly, in each tiller, there were leaves at different stages of development (growing/fully expanded/senescence). When a leaf became fully expanded (ligule exposed) and completely green, it was considered as 1 leaf unit; when the leaf started to senesce until it was completely senesced, it was considered 0.5 leaf unit; when the leaf was still expanding, it was considered 0.5 leaf units and, finally, all leaf units were summed up. The total number of leaves per tiller was calculated at regular intervals throughout the experiment; however, Ln data were considered only as the leaf amount attached to tillers just before each harvest. Leaf lifespan (LLS; dd) was determined as the product of the average phyllochron and maximum number of leaves per tiller.

Before each harvest, two samples per plot (0.24 m^2^ each) were cut at ground level, taken to the laboratory, the number of live tillers was counted, and tiller population density (TPD) was expressed in tiller/m^2^. Afterwards, the leaves of two samples of 50 tillers were detached from the ligule, and their lengths (cm) and areas (cm^2^) were measured using a leaf area meter (LAI-3100C; LI-COR Inc., Lincoln, Nebraska). Each leaf was dried in an oven at 65°C for at least 72 h and then weighed on a precision scale. Specific leaf area (SLA; cm^2^ g^-1^) was calculated as the ratio of the sum of individual leaf areas to the dry weight. The correction factor (FC; g cm^-1^) was determined based on the relationship between the length of each leaf and its dry weight. The leaf weight ratio (LWR) was obtained by calculating the relationship between the leaf dry weight and plant dry weight (combined leaf and stem + pseudostem dry weight). Leaf area per tiller (LA) was measured by dividing the total leaf area measured in each sample by the number of tillers in this sample.

Additionally, when the defoliation criteria were reached (canopy height: 20 cm), two 0.7m × 0.2 m samples (1.4 m^2^), per experimental unit, were cut at ground level. The samples were taken to the laboratory, dried in an oven at 65 ˚C for 72 h, and weighed (Dw_i_). At the same time, two other 0.7m × 0.2m (1.4 m^2^) areas, per experimental unit, in conditions similar to those previously sampled were set and cut at 10 cm of height and the forage inside the quadrat taken to the laboratory, dried in an oven at 65 ˚C for 72 h and weighted (Dw_f_). Forage production in each cycle (kg ha^-1^) was estimated by subtracting the herbage mass existing at a given pre-cutting stage (and expressed in kg of DM per ha) from the residual (after cut) herbage mass of the previous post-cutting forage mass (both cut at the ground level). The annual forage accumulation was determined by summing the average forage biomass per cycle throughout the year (kg ha^-1^). The relative growth rate (RGR; kg kg^-1^ dd^-1^) per m^2^ was calculated using the formula [ln(Dw_f_)–ln(Dw_i_)] × (dd cycle^−1^)^−1^ [45], and the relative growth rate per plant (RGR plant^-1^) was calculated. In addition, canopy density (CD; kg m^-1^ ha^-1^) was measured as the ratio of dry matter before cutting (Dwi) to canopy height (0.2 m). The weight per tiller (WT; g tiller^-1^) was calculated by dividing the total plant dry weight by the number of tillers per unit area.

### Statistical analysis

Observations from each species (treatment) throughout the year were analyzed by cycle and per year, depending on the variable being measured. Cutting events were based on height criteria and cutting days were not performed simultaneously for either species during the experimental period. Additionally, the measurement intervals in the field were not uniform; therefore, the thermal time was used to standardize the comparisons.

**Fig 3:**
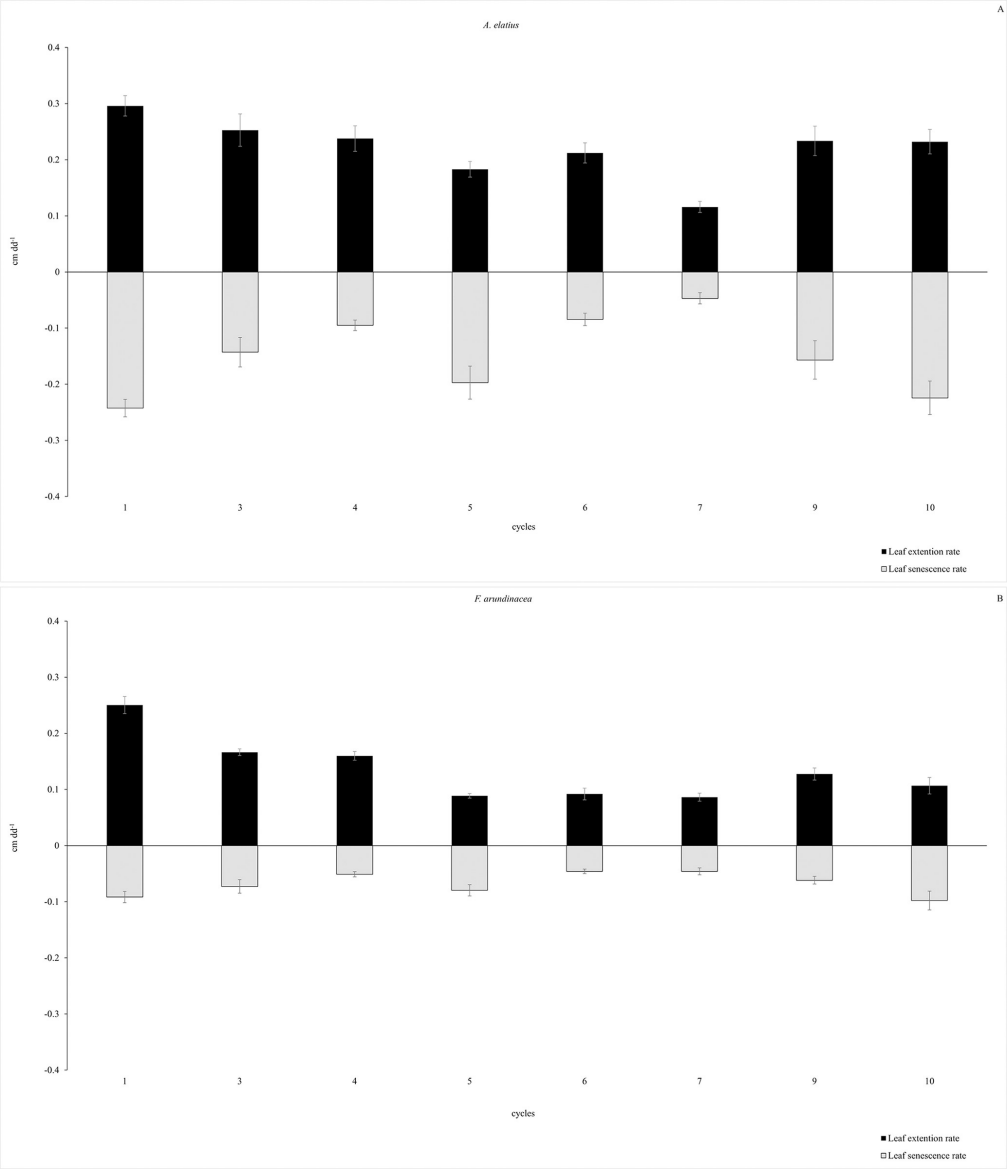
LER (cm dd^-1^) (black bars) and LSR (cm dd^-1^) (gray bars) per plant throughout the cycles in (A) *A*. *elatiu*s and (B) *F*. *arundinacea*. Results are presented as mean ± standard error (n = 57).

**Fig 4:**
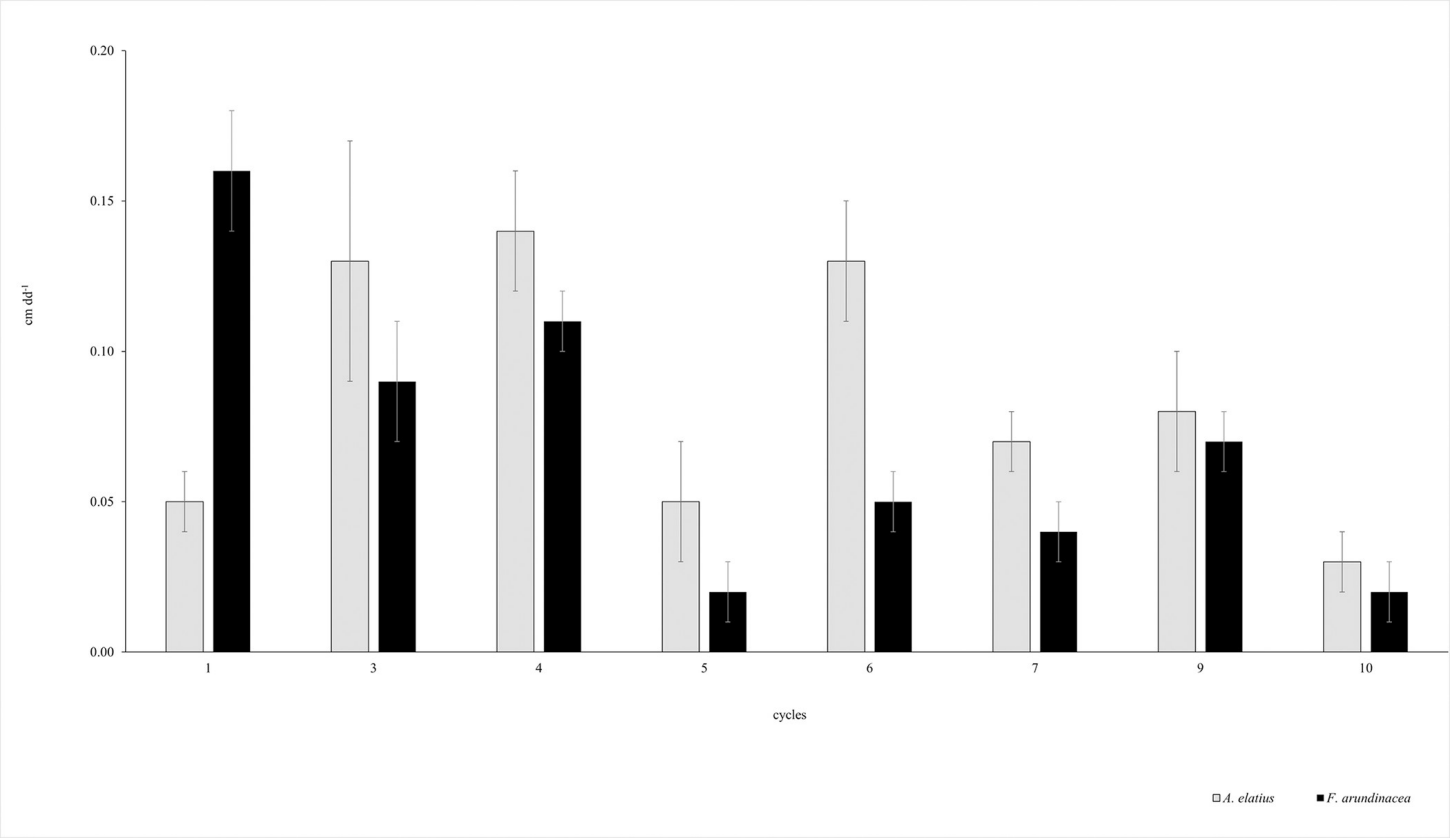
BLG (cm dd^-1^) throughout the cycles in *A*. *elatiu*s (gray bars) and *F*. *arundinacea* (black bars). Results are presented as the mean ± standard error (n = 60).

Data were analyzed using analysis of variance (ANOVA) in the RStudio Team [46]. The statistical model was Y_[ij]_ = μ +α_[i]_ +ϵ_[ij]_; where, Y_[ii]_ = random variable corresponding to _j-th_ observation of the _i-th_ treatment; μ = constant effect or global average; α_[i]_ = effect of treatment i-th treatment; and ϵ_[i]j_ = error term. Depending on the objective of the analysis, *A*. *elatius* and *F*. *arundinacea* were considered as treatments (one fix effect, results Table 1). On the other hand, cycle was also considered as treatment when appropriate. Each species was analyzed separately (one fix effect, results Table 2 and Figs 3A, 3B and 4). Before conducting the analysis, the assumption of normality was verified. If the assumptions were not met, that is, if there was evidence of a pattern or association in our data, a randomized linear model (Null Model) was used [47] instead of a non-parametric approach. In this case, the F-value obtained from the Analysis of Variance (ANOVA) was used to assess the strength of the effect of the independent variable on the dependent variable. To determine the significance of the effect, the original F-value was compared with the distribution of F-values generated by the permutation of the original data. Specifically, 10,000 permuted matrices were created and compared with the original data. If the observed difference in the F-value was statistically significant, the null model would show differences of less than 0.0001. This suggests that the independent variable has a significant effect on the dependent variable, which cannot be explained exclusively by random variation.

**Table 1:** Descriptive metrics values for leaf and community traits for *A*. *elatius* and *F*. *arundinacea*.

Traits	n	*A*. *elatius*	*F*. *arundinacea*	P-value
		Media±S.E	Media±S.E	
SLA (cm^2^ g^-1^)	41	269.33±6.99	145.15±5.30	<0.001
LA (cm^2^)	48	13.09±0.52	9.87±0.43	<0.001
LER (cm dd^-1^)	48	0.2203±0.0099	0.1347±0.0083	<0.001
LSR (cm dd^-1^)	48	-0.1487±0.0121	-0.0683±0.0042	<0.001
BLG (cm dd^-1^)	48	0.0897±0.0091	0.0722±0.0080	0.093
LLS (dd)	48	277.34±11.94	461.39±14.88	<0.001
Ln	47	3.26±0.11	2.53±0.06	<0.001
Ph (dd^-1^)	47	97.26±2.47	208.15±7.38	<0.001
LWR (g g^-1^)	47	0.64±0.02	0.74±0.01	<0.001
FC (g cm^-1^)	41	0.0015±0.0002	0.002±0.000038	<0.001
CD (kg m^-1^ha^-1^)	48	13498.07±427.63	20219.79±601.64	<0.001
WT (g tiller^-1^)	48	0.63±0.02	1.05±0.03	<0.001
TPD (tiller m^-2^)	48	2648.77±129.15	3444.48±159.82	<0.001
TER (cm dd^-1^)	46	0.05±0.0027	0.04±0.0021	0.248
F. mass (Kg ha^-1^)	46	982.847±78.92	1221.378±116.69	0.0656
RGR (kg kg^-1^ dd^-1^)	45	0.001752±0.0001	0.001473±0.0001	0.0836

Mean ± standard error (S.E) of specific leaf area (SLA); leaf area per tiller (LA); leaf elongation rate (LER); leaf senescence rate (LSR); leaf life span (LLS); balance of leaf growth (BLG); leaf life span (LLS); number of leaves (Ln); phyllocrone (Ph); leaf weight rate (LWR); leaf weight/length (FC); canopy density (CD); weight per tiller (WT); tiller population density (TPD); tiller elongation rate (TER); forage mass (F. mass); relative growth rate (RGR).

**Table 2:** Coefficient of variation (C.V.) of leaf functional and forage productivity traits in *A*. *elatiu*s and *F*. *arundinacea* throughout the year.

Traits	Species	%
LER	*A*. *elatius*	31.19
	*F*. *arundinacea*	43.03
LSR	*A*. *elatius*	56.42
	*F*. *arundinacea*	42.66
Forage mass	*A*. *elatius*	54.46
	*F*. *arundinacea*	51.66
RGR	*A*. *elatius*	58.34
	*F*. *arundinacea*	81.43

Traits: LER (cm dd^-1^), LSR (cm dd^-1^), forage biomass accumulation (kg ha^-1^), and RGR (kg kg^-1^ dd^-1^). The results are presented as percentages (n = 48).

**Fig 1:**
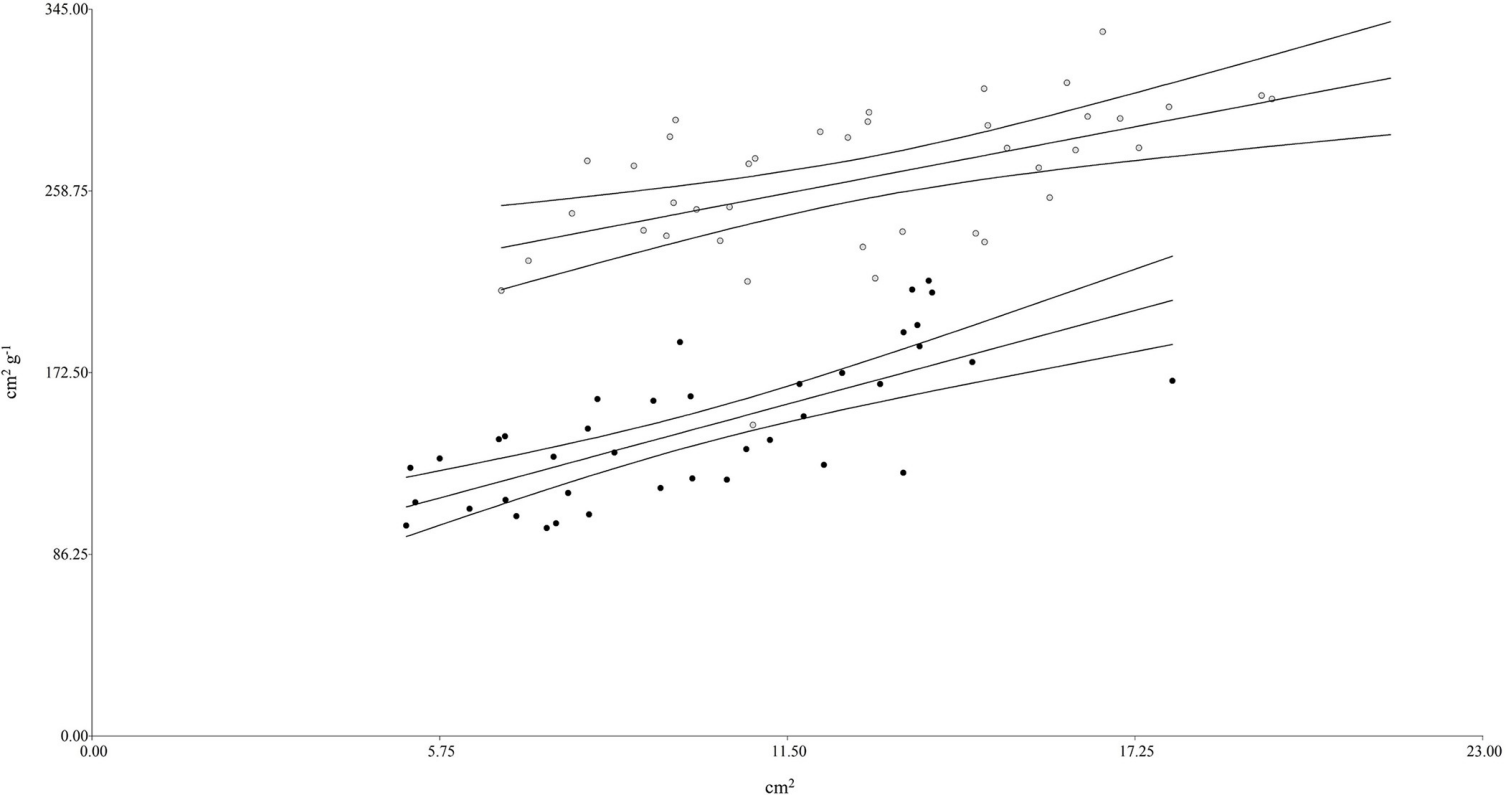
Linear regression curve and confidence bands (95%) for the relationship between SLA (cm^2^ g^-1^) and LA (cm^2^ g^-1^) on *A*. *elatius* and *F*. *arundinacea* throughout the year. Linear regression parameters: *A*. *elatius*, y = 5.47x + 194.83; R^2^ = 0.28; P < 0.0004; *F*. *arundinacea*, y = 7.74x + 68.56; R^2^ = 0.52; P <0.0001.

**Fig 2:**
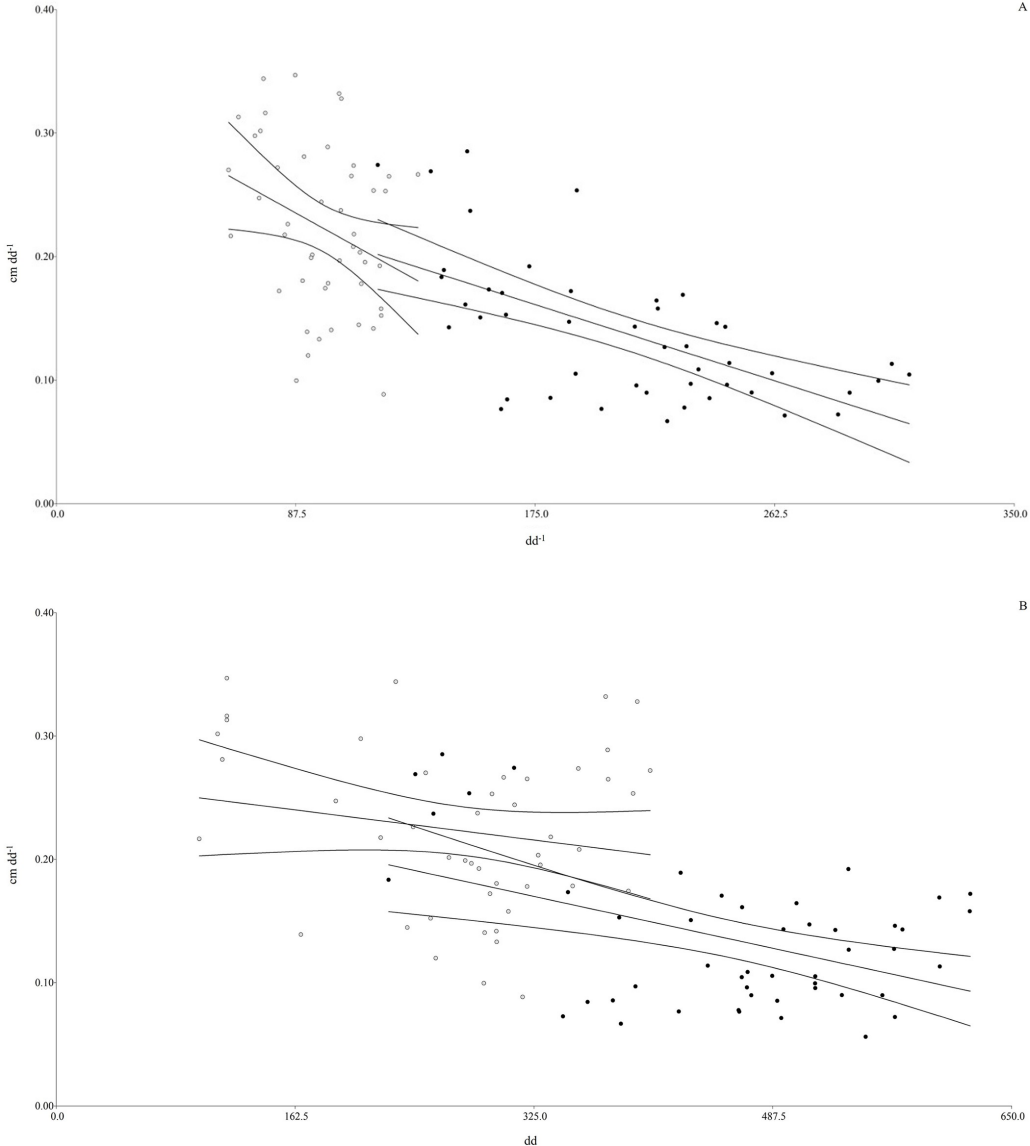
**(A) Linear regression curve and confidence bands (95%) of the relationship between LER (cm dd**^**-1**^**) and Ph (dd**^**-1**^**) in *A*. *elatius* and *F*. *arundinacea* throughout the year.** Linear regression parameters: *A*. *elatius*, y = -0.0023 x + 0.34; R^2^ = 0.09; P <0.03; *F*. *arundinacea* y = -0.0007x + 0.28: R^2^ = 0.35; P <0.0001. **(B) Linear regression curve and confidence bands (95%) of the relationship between LER (cm dd**^**-1**^**) and LLS (dd) on *A*. *elatius* and *F*. *arundinacea* throughout the year.** Regression parameters: *A*. *elatius* y = -0.00015x + 0.26; R^2^ = 0.03; P = 0.2; *F*. *arundinacea* y = -0.0002x + 0.25; R^2^ = 0.21; P <0.0009.

Linear regression analysis was conducted using Infostat [48] to explore the relationships between variables. The statistical lineal model was Y_[i]_ = β_0_+β_1_X_[i]_+ϵ_[i]_; where, Y_[i] =_ variable response (dependent variable); β_0_ = intercept of the lineal regression, β_1_X_[i]_ = slope of change in variable (independent variable); and ϵ_[i]_ = error term. The objective of this analysis (Figs 1 and 2A, 2B) was to identify similarities in behavior throughout the year among species. Instead, we performed a t-test to compare the slopes and intercepts between regressions with a significance level of p < 0.05, in the RStudio Team [46].

Additionally, multivariate canonical correlation analysis (CCA) was performed separately for each species by the RStudio Team [46]. The analysis aimed to explore the relationship between two groups of variables: the independent group, which included some species variables such as SLA, LA, LER, LSR, LWR, Ph, Ln, LLS, WT, CD, and TPD, and the dependent group, which included forage productivity parameters (kg ha^-1^ and RGR).

## Results

### Patterns at plant-scale and canopy structure

*A*. *elatius* and *F*. *arundinacea* exhibited significant differences in functional traits at the plant scale and in demographic parameters (Table 1). Specifically, *A*. *elatius* had almost two-fold higher LER and LSR rates than *F*. *arundinacea*. Both species had similar forage mass, RGR, and TER.

*Festuca arundinacea* had higher Ph and LLS values than *A*. *elatius*. However, *A*. *elatius* had more Ln and lighter leaves (lower LWR and FC, and greater SLA) than *F*. *arundinacea* also, a lower WT. A greater LA was observed in *A*. *elatius* than in *F*. *arundinacea*; however, the latter showed higher TPD and canopy density (CD).

### Relationship between plant functional traits and leaf growth rate across the year

There was a positive relationship between the dependent trait SLA and LA throughout the experimental period for both species (Fig 1). This relationship was significantly correlated across both species and showed a similar pattern. Specifically, both species experienced a general increase in SLA with a higher LA, and their regressions exhibited similar tendencies. However, the regression for each species showed a different SLA-intercept (P <0.02) and the same slope (P = 0.20) throughout the year.

Additionally, a similar significant relationship was observed for both species when the LER was plotted against LLS and Ph throughout the experimental period (Fig 2A and 2B). A negative relationship between leaf elongation rate per plant and leaf lifespan was observed (R^2^ = 0.46; P <0.0001), as was the case with LLS (R^2^ = 0.35, P <0.0001). Both species showed the same response patterns, with a higher LER related to lower Ph and LLS (Fig 2A, test-t: intercept: P >0.44, and slope: P > 0.46. Fig 2B, test-t: intercept: P >0.85; slope: P >0.51). *A*. *elatius* was related to a higher LER and lower Ph and LLS than *F*. *arundinacea* (Table 1).

### Leaf growth and senescent events through cycles

Although significant differences in leaf growth were observed between cycles in grasses (P <0.0001), *A*. *elatius* had the highest LER and LSR in all cycles compared to *Festuca* (Fig 3). Similar intra-annual stability in LER and LSR throughout the experimental period was observed between grasses; that is, both grasses had similar coefficients of variation (Table 2). Furthermore, both grasses shared the same distribution of leaf growth rates throughout cycles; cycles 1, 3, 4, 9, and 10 showed higher rates than the others in both species. Moreover, a comparable distribution of leaf senescence rates through the cycles was observed. In addition, the balance of leaf growth per year was the same in both species (Table 1), and among the cycles, both exhibited the same leaf growth balance in cycles 3, 4, 5, 9, and 10 (Fig 4).

### Forage productivity and growth analysis throughout cycles

**Fig 5:**
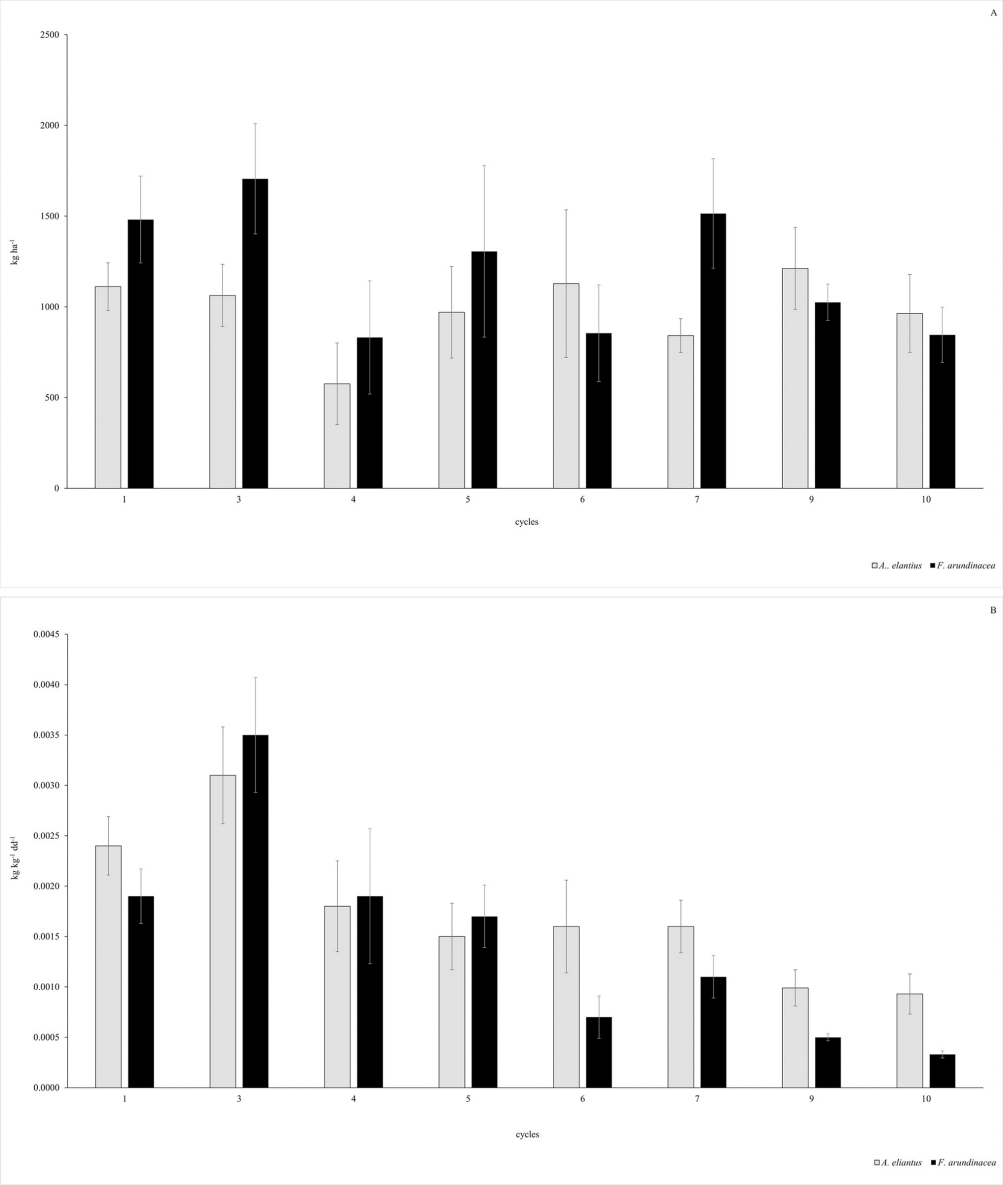
**(A) Forage biomass accumulation (kg ha**^**-1**^**) throughout the cycles for *A*. *elatiu*s and *F*. *arundinacea***, and **(B) RGR (kg kg**^**-1**^ **dd**^**-1**^**) throughout the cycles for *A*. *elatiu*s and *F*. *arundinacea*.** The results are presented as mean ± standard error (n = 6).

At canopy level, the annual forage biomass accumulation was 7863.26 kg ha^-1^ for *A*. *elatius* and 9564.73 kg ha^-1^ for *F*. *arundinacea;* throughout the year similar forage mass average was observed (Table 1), and the same inter-annual stability was observed between the grasses (C.V._*A*. *elatius*_: 54.46, C.V._*F*. *arundinacea*_: 51.66; Table 2). Although different seasonal accumulations were observed, *F*. *arundinacea* showed a higher forage biomass accumulation during spring/autumn/winter (cycles 3>7>1>5>9>6>10>4), whereas *A*. *elatius* was more productive during autumn/spring/winter (cycles 9>6>1>3>5>10>7>4), whereas a similar RGR between grasses was observed (Table 1) and shared the same distribution of RGR through cycles (Fig 5), as was observed previously in LER and LSR functional traits (Fig 3). However, *F*. *arundinacea* (Table 2) showed lower RGR stability than *A*. *elatius*.

At the plant scale, *A*. *elatius* exhibited a higher rate of RGR plant^-1^ (μ = 3.3^E-10^ kg kg^-1^ dd^-1^ ± S.E. = 2.8^E-11^) than *F*. *arundinacea* (μ = 2^E-10^ kg kg^-1^ dd^-1^ ± S.E. = 2.2^E-11^).

### Correlation of forage productivity and plant and canopy features through cycles

The structure of the relationship between forage productivity (kg/ha and RGR) and functional traits at the tiller and canopy structure (SLA, LA, LER, LSR, LWR, Ph, Ln, LLS, TDP, W, and CD) was analyzed using canonical correlation analysis (CCA). The analysis was performed separately for each species to observe differences between them. Two significant canonical correlations were observed in *A*. *elatius* (Table 3). The first canonical correlation had the highest value (0.919), accounting for 84% of the variance (L1). The second canonical correlation coefficient was 0.765, which explained 58.5% of the variance. The coefficients in the linear combinations reflect the contribution of each trait to canonical correlation. The first canonical correlation showed higher vectors for LLS and Ln with kg ha^-1^ and RGR, respectively. In L2 (which explained less of the variance), both forage production attributes (kg ha^-1^ and RGR) had similar vectors, and were related to LLS, Ln, CD, LWR, and TDP (Table 3).

**Table 3:** Canonical correlations and coefficients of linear combination products of multivariate canonical correlation analysis between forage productivity traits and functional and canopy structure traits in A. *elatius* and *F*. *arundinacea*.

	*A*. *elatius*	* *	*F*. *arundinacea*	
**Canonical correlations**	L(1)	L(2)	L(1)	L(2)
R	0.919	0.765	0.8813	0.447
R^2^	0.844	0.585	0.776	0.2
p-value	<0.001	<0.001	<0.001	ns
**Coefficients of linear combinations**	L(1)	L(2)	L(1)	L(2)
SLA	-0.163	-0.052	-0.073	-0.517
LA	0.01	-0.069	-0.193	0.343
LER	0.12	0.391	0.057	0.32
LSR	-0.246	-0.024	0.063	-0.117
LWR	-0.115	-0.611	0.057	0.204
Ph	-0.177	0.212	-0.105	1.04
Ln	-1.73	0.921	0.324	0.386
LLS	2.1	-1.39	-0.874	-1.01
TPD	-0.092	0.58	0.245	0.604
WT	-0.317	-0.486	1.29	-2.35
CD	0.782	0.818	-1.24	2.46
Kg ha^-1^	1.17	0.47	-0.846	0.93
RGR	-1.08	0.641	1.2	0.09

The forage productivity traits: Kg ha-1 and RGR; the functional and canopy structure traits: SLA, LA, LER, LSR, LWR, Ph, Ln, LLS, TDP, WT, CD.

For *F*. *arundinacea*, there was a significant canonical correlation between the traits (0.881, explaining 77.6% of the variance). The coefficients of the linear combinations of functional traits in the first canonical correlation showed a higher contribution between LLS and CD with Kg ha^-1^; RGR was related to WT (Table 3); that is, the productivity (kg/ha and RGR) in *F*. *arundinacea* was related to plant function at the tiller and canopy levels.

## Discussion

In a fertile environment subjected to frequent and moderate defoliation, *F*. *arundinacea* and *A*. *elatius* exhibited distinct intrinsic traits (Table 1 and Fig 1). *F*. *arundinacea* had a denser canopy structure and *A*. *elatius* invested more in tissue renovation (higher LER, LSR, Ln and LA). *A*. *elatius* exhibited characteristics typical of fast-growing, resource-acquisitive grasses, whereas *F*. *arundinacea* displayed traits associated with more conservative grass species. Despite this, both grasses had similar relative growth rates (kg kg^-1^ dd^-1^) and net balance of leaf growth (Table 1 and Figs 4 and 5) and presented a similar distribution of leaf growth throughout the year (Table 2 and Figs 3 and 4). These differences result mainly from contrasting growth strategies [3, 5, 7, 8, 49]. In this sense, both grasses differ in how they partition nutrient supplies [2] and are clearly situated at different positions along the ecological spectrum of resource acquisition and use (Table 1). In this context, *A*. *elatius* is clearly classified as a fast-growing, resource-acquisitive plant, whereas *F*. *arundinacea* is a slow-growing, more conservative grass. However, both grasses exhibited a trade-off between plant functional traits and demographic parameters that produced similar results in terms of forage production.

### Some ecological remarks on species differences

A higher rate of new leaf tissue production (LER and LAR) in *A*. *elatius* than in *F*. *arundinacea* was consistent with the different growth strategies among species, as described by Arredondo and Schnyder [19]. Additionally, a higher rate of leaf senescence (LSR) was observed in *A*. *elatius* (Table 1 and Fig 3), which is consistent with recent findings on senescent leaf traits in fast-growing grasses in natural ecosystems [50]. In most of the evaluated cycles, coordination and synchronization between the LER and LSR events were observed in both species, except in cycles 1 and 6 (Fig 4). This association between leaf developmental events in plants has been recognized and explained by several authors [6, 51–54]. However, *A*. *elatius* exhibited faster coordination than *F*. *arundinacea*, which was related to its greater ability to utilize available resources and renew tissues.

Both species exhibited a significant positive relationship between SLA and LA throughout the year, with a similar slope (*P*<0.0001; data not show); however, the relationship between the two species was different. Specifically, at a similar LA, *A*. *elatius* consistently had a higher SLA than *F*. *arundinacea*. Additionally, the intercept for *A*. *elatius* was higher than that of the slow-growing species (2.54 times), indicating a contrasting growth strategy between the two species (Fig 1). Comparable relationships have been observed between RGR and SLA in plants with different life cycles and growth strategies of the genus Physaria [55]. However, these authors did not find a clear relationship between leaf morphology and functioning, as would be expected according to the leaf economic spectrum theory and proposed that the patterns of leaf structure and function may not be as universal as previously thought.

On the other hand, a significant relationship between LLS and Ph was observed throughout the year in both *F*. *arundinacea* and *A*. *elatius* (*P*<0.0001; data not show). Also, a similar relationship between LLS and Ph was observed in both grasses (Fig 2A and 2B). Although our relationship was weaker than that reported in the literature (with smaller R^2^ values), this could be attributed to differences in data scale as well as varying growth seasonality and smaller differences in slopes between species. Conversely, in the canopy structure, the smaller significant relationship between LLS and RGR (results not shown) was consistent with the findings of Diemer et al. [56], who showed a weaker association between LLS and light-use efficiency and, consequently, the accumulation of aboveground biomass in 29 perennial herbaceous species. Reich et al. [24] reported a positive association between LLS and other plant functional traits, which reflect a set of mutually supporting traits that interact to determine plant behavior and production and the community’s ability to retain carbon in its biomass [57].

### Different growth strategies and the same forage productivity

The previous results allowed us to categorize the model species into contrasting growth strategies. However, we discovered some interesting productivity similarities, such as a similar RGR, and forage biomass accumulation (Table 1 and Figs 4 and 5). This raises the question of how slow-growing grasses can achieve productivity similar to that of fast-growing grasses in fertile environments. What compensatory changes enable slow-growing grasses to be as productive as fast-growing ones? To answer these questions, we divided forage production into two categories: plant functional traits and demographic parameters.

At specific thermal times, *A*. *elatius* and *F*. *arundinacea* exhibited different leaf growth and senescence rates as previously described. However, the balance between these rates was similar throughout the year (BLG in Table 1), which could play an essential role in forage production. Skinner and Nelson [51] linked the forage production with LER, but our results did not substantiate such a relationship. Similarly, higher LWR (g g^-1^), FC (g cm^-1^), and tiller weight (WT; g tiller^-1^) in *F*. *arundinacea* than *in A*. *elatius* could partially compensate for other functional traits, such as lower LER, LA, and leaf number, which are useful indicators of potential productivity [35, 58]. At the canopy scale, *F*. *arundinacea* had higher TPD and CD (Table 1) as well as higher tiller survival rates (TSR) [28]. Garay et al. [59] postulated that herbage production can be attributed to a combination of tiller density and WT, with increases in either or both factors leading to an increase in primary forage growth. *F*. *Arundinacea* was demonstrated in a fertile environment, and under moderate and frequent defoliation, it had attributes that buffered the expected higher net dry weight forage pruduction of *A*. *elatius* and could compensate for the slower RGR plant^-1^ ratio.

Multivariate canonical correlation analysis was conducted for each grass species to determine the associations between the two groups of variables [60], which allowed us to identify distinct associations between plant functional traits at the tiller and canopy structure levels, and productivity traits. The independent variables were functional traits at the plant and canopy levels and the dependent variables were forage productivity traits (kg ha^-1^ and RGR). Although the linear coefficients differed between the plant- and community-level attributes, both grass species exhibited significant correlations between the two variable groups (Table 3). In *A*. *elatius*, grass productivity was mainly correlated with plant-level functional traits, such as LLS and Ln, whereas *F*. *arundinacea* had a high correlation between forage productivity and demographic parameters at the canopy level (CD and WT) and plant functional trait LLS.

## Conclusion

In conclusion, in a fertile soil environment, some so-called negative characteristics for growth (such as higher pH, lower LER, and SLA) in perennial slow-growing species are related to the specific time scale and could be compensated by demographic parameters to allow forage biomass accumulation similar to that observed in fast-growing species. Leaf growth traits alone should be used with caution to make sense of productivity of grasses, and other traits, such as leaf senescence rate and LWR, should also be considered. In addition, these factors should be considered along with demographic parameters to explain forage production.

*F*. *arundinacea* had some competitive advantages coming from the population level, which allowed it to have similar relative growth rate and accumulated forage as fast-growing *A*. *elatius*, in most cycles. The relationships between annual productivity and SLA, LWR, Ph, and LER should be carefully used to make inferences about the productivity of slow-growing grasses. Similarly, it seems that SLA may not be considered the leading indicator for determining interspecific variation in RGR, and that the net forage production in *F*. *arundinacea* showed a higher correlation with more attributes than *A*. *elatius*. This supports the idea of higher plasticity in terms of forage productivity in *F*. *arundinacea*, as expected in slow-growing species.

## Supporting information

S1 Table Climate variables throughout the data collection period in the experimental area. Mean rainfall (mm), mean minimum temperature (T) Min,°C), mean ± standard error (S.E), mean temperature (T. Mean,°C), and mean maximum temperature (T. Max, ±C), hour of insolation (Ins, h). Historical mean values of rainfall and temperature for the last 85 years in Lages, Santa Catarina, Brazil.
(PDF)

S2 Table Data collection per cycle. Data from June 2014 to April 2015 per cycle in each experimental unit for *A*. *elantus* and *F*. *Arundinacea*.
(PDF)
