# Supplementary material for: Unraveling the forage productivity puzzle: Comparing fast and slow-growing grasses
Source: PLoS One. 2024 Jul 30;19(7):e0306692. doi: 10.1371/journal.pone.0306692 (PMC11288426; doi:10.1371/journal.pone.0306692)
Supplement: S1 Table — Mean rainfall (mm), mean minimum temperature (T) Min,°C), mean ± standard error (S.E), mean temperature (T. Mean,°C), and mean maximum temperature (T. Max, ±C), hour of insolation (Ins, h). Historical mean values of rainfall and temperature for the last 85 years in Lages, Santa Catarina, Brazil. (PDF) [file pone.0306692.s001.pdf]

S1 Table. Climate variables throughout the data collection period in the experimental area.

Monthly mean value						Historical mean value	
Month- year	Rainfall (mm)	T. Min(°C)	T. Max (°C)	T. Mean Media±S.E	Ins (hours)	Rainfall (mm)	Temp (°C)
June-2014	397.8	8.1	16.4	12.0 ±0.57	85.7	107.1	11.4
July-2014	77.3	6.6	16.6	11.3± 0.53	106.7	114.7	11.0
August-2014	61.0	6.3	20.6	12.6± 0.67	204.7	123.9	12.2
September-2014	233.4	10.5	20.6	15.1± 0.45	120.2	148.1	13.5
October-2014	154.2	12.2	24.0	17.3± 0.55	184.3	172.9	15.6
November-2014	194.8	13.2	23.9	18.1± 0.29	172.8	122.0	17.4
December-2014	187.2	14.7	25.3	19.5± 0.34	133.1	131.1	19.3
January-2015	211.9	15.7	27.3	20.8± 0.34	197.0	158.1	20.4
February-2015	133.9	14.3	24.8	18.8± 0.29	163.0	146.9	20.2
March- 2015	135.6	14.6	25.3	19.1± 0.32	174.8	117.0	19.1
April- 2015	99.7	12.1	22.2	16.4± 0.33	147.9	103.3	16.3
May- 2015	71.0	10.2	18.7	13.9± 0.43	106.7	107.4	13.2
June-2015	134.9	6.4	17.4	11.4± 0.56	127.4	107.1	11.4

Mean rainfall (mm), mean minimum temperature (T) Min, °C), mean ± standard error (S.E), mean temperature (T. Mean, °C), and mean maximum temperature (T. Max, °C), hour of insolation (Ins, h). Historical mean values of rainfall and temperature for the last 85 years in Lages, Santa Catarina, Brazil.
