# Supplementary material for: Unraveling the forage productivity puzzle: Comparing fast and slow-growing grasses
Source: PLoS One. 2024 Jul 30;19(7):e0306692. doi: 10.1371/journal.pone.0306692 (PMC11288426; doi:10.1371/journal.pone.0306692)
Supplement: S2 Table — Data from June 2014 to April 2015 per cycle in each experimental unit for A. elantus and F. Arundinacea. (PDF) [file pone.0306692.s002.pdf]

S2 Table. Data collection per cycle

	A. elantus _1			*A. elantus* _2			*A. elantus* _3			*F. Arundinacea* _1, _2, _3		
**Cycle**	**Start**	**Finish**	**Days**	**Start**	**Finish**	**Days**	**Start**	**Finish**	**Days**	**Start**	**Finish**	**Days**
1	04/06/2014	09/07/2014	35	04/06/2014	09/07/2014	35	04/06/2014	09/07/2014	35	04/06/2014	09/07/2014	35
3	06/08/2014	25/08/2014	19	06/08/2014	25/08/2014	19	06/08/2014	25/08/2014	19	06/08/2014	25/08/2014	19
4	01/09/2014	20/09/2014	19	01/09/2014	20/09/2014	19	01/09/2014	20/09/2014	19	01/09/2014	20/09/2014	19
5	23/09/2014	15/10/2014	22	23/09/2014	15/10/2014	22	23/09/2014	15/10/2014	22	23/09/2014	15/10/2014	22
6	24/10/2014	13/11/2014	20	24/10/2014	13/11/2014	20	24/10/2014	13/11/2014	20	24/10/2014	13/11/2014	20
7	21/11/2014	08/12/2014	17	21/11/2014	08/12/2014	17	21/11/2014	08/12/2014	17	21/11/2014	17/12/2014	26
9	21/01/2015	18/02/2015	28	21/01/2015	25/02/2015	35	21/01/2015	25/02/2015	35	21/01/2015	25/02/2015	35
10	25/02/2015	08/04/2015	42	05/03/2015	30/04/2015	56	05/03/2015	30/04/2015	56	05/03/2015	30/04/2015	56

Data from June 2014 to April 2015 per cycle in each experimental unit for *A. elantus* and *F. Arundinacea*.
